# (Pro)renin Receptor Expression Increases throughout the Colorectal Adenoma—Adenocarcinoma Sequence and It Is Associated with Worse Colorectal Cancer Prognosis

**DOI:** 10.3390/cancers11060881

**Published:** 2019-06-24

**Authors:** Maider Beitia, Jon Danel Solano-Iturri, Peio Errarte, Julio Calvete-Candenas, Alberto Loizate, Mari Carmen Etxezarraga, Begoña Sanz, Gorka Larrinaga

**Affiliations:** 1 Department of Physiology, Medicine and Nursing Faculty, University of the Basque Country (UPV/EHU), Leioa, 48940 Bizkaia, Spain; maider.beitia@ehu.eus (M.B.); peio.errarate@ehu.eus (P.E.); mariabegona.sanz@ehu.eus (B.S.); 2 Department of Nursing, Medicine and Nursing Faculty, University of the Basque Country (UPV/EHU), Leioa, 48940 Bizkaia, Spain; 3 BioCruces Research Institute, Barakaldo, 48903 Bizkaia, Spain; jondanel.solanoiturri@osakidetza.eus (J.D.S.-I.); mariacarmen.etxezarragazuluaga@osakidetza.eus (M.C.E.); 4 Department of Pathology, Cruces University Hospital, Bilbao, 48903 Bizkaia, Spain; 5 Service of Oncology, University Hospital Puerta del Mar, 11009 Cádiz, Spain; jjcalvete@gmail.com; 6 Department of Surgery, Basurto University Hospital, University of the Basque Country (UPV/EHU) Bilbao, 48013 Bizkaia, Spain; alberto.loizatetotoricaguena@osakidetza.eus; 7 Department of Anatomic Pathology, Basurto University Hospital, University of the Basque Country (UPV/EHU), Bilbao, 48013 Bizkaia, Spain

**Keywords:** (pro)renin receptor, adenoma, colorectal cancer, biomarkers, angiotensin, Wnt, vATPase

## Abstract

(Pro)renin receptor (PRR) is a protein that takes part in several signaling pathways such as Renin Angiotensin System and Wnt signalling. Its biological role has recently been related to cancer progression and in this study, we investigated its relevance in colorectal cancer (CRC). To that end, we analysed the immunohistochemical expression of PRR in adenomatous polyps and CRCs from the same patients (*n* = 42), and in primary tumours and nodal and liver metastases from advanced CRC patients (*n* = 294). In addition, the soluble fraction of PRR was measured by ELISA in plasma samples from 161 CRC patients. The results showed that PRR expression was gradually augmented along the uninvolved mucosa–adenoma–adenocarcinoma sequence. Besides, the stronger expression of PRR in primary tumours was markedly associated with local tumour extent and the onset of metastases. Moreover, PRR expression in both primary and distant metastases was associated with worse 5- and 10-year survival of CRC patients. Plasmatic PRR levels did not change with respect to controls and were not associated with CRC aggressiveness. These results suggest a key role of PRR in the development and progression of CRC and a potential use of this protein as a new prognostic biomarker and/or therapeutic target for this disease.

## 1. Introduction

Colorectal cancer (CRC) is one of the most common cancers worldwide, considerably increased in Western countries for its association with sedentary lifestyles and bad diet habits [1]. Even if the lately implemented screenings for its early detection have diminished the death rates, its high incidence still makes it the second cause of cancer-related deaths [2]. Therefore, a better understanding of the biomolecular changes involving colorectal carcinogenesis is necessary for the design of effective diagnostic, prognostic and therapeutic tools.

(Pro)renin receptor (PRR) was discovered in 2002 by Nguyen et al. [3]. It is a component of the Renin Angiotensin System (RAS), an endocrine peptide system classically related to the regulation of blood pressure and hydro-electrolytic balance [4]. The main physiological functions of RAS seem to be transduced by the angiotensin II type 1 receptor (AT1R) pathway [5,6,7,8,9,10,11,12,13]. However, beyond its classically known functions, RAS has been proved to be a complex system expressed in several tissues whose bioactive peptides and receptors locally regulate processes such as cell growth and proliferation too. In the last two decades, there has been an exponential increase in epidemiologic, basic and translational research demonstrating that imbalances in RAS are associated with cancer development and progression [7,14,15].

PRR is a protein able to bind renin enzyme and its inactive precursor prorenin, causing the activation and increase in their activity. Active renin catalyses the conversion of angiotensinogen into angiotensin I, contributing to the generation of angiotensin peptides that will further trigger the consequent effects by its binding to their receptors [16,17]. Moreover, PRR also acts as a membrane receptor which, after (pro)renin binding, is able to activate different intracellular secondary messengers such as MAPK, ERK1/2, TGF-B, PLZF, PAI-1 or COX2 [3,18,19,20,21,22]. 

Besides, PRR has also been associated with the Wnt–β-catenin signalling pathway [23], the activation of which is an initiating event of colorectal carcinogenesis [24,25]. Additionally, PRR has been tightly linked to the vacuolar ATPase (vATPase). In fact, it is an accessory protein of the vATPase that seems to be essential for its correct assembly [3,26]. vATPase activity has been linked to cancer, as it is associated with compartment acidification and autophagy, among others functions [27].

Moreover, PRR can be found in different conformations: full-length or truncated. In fact, both furin and ADAM19 enzymes are able to cleave PRR, generating a truncated membrane protein and a soluble PRR (sPRR) [28,29,30]. The latter can be found in human fluids such as blood or urine, which could be used as biomarker for detection or prognosis of different diseases [28]. 

Several preliminary studies suggest an important role of PRR in cancer cells, where this protein has been found overexpressed [31,32,33,34,35,36]. Moreover, this overexpression is able to alter the hallmarks of cancer described by Hannahan and Weinberg [37] by different mechanisms. In fact, PRR can encourage proliferation and inhibit apoptosis by the activation of MAPK and PI3K/AKT intracellular pathways [31,34]. Additionally, its tumorigenic role has been associated with the activation of intracellular signalling stimulated by (pro)renin binding, as well as to vATPase and Wnt–β-catenin signalling pathways [33,35,36]. Regarding the latter, a very recent study has demonstrated that PRR can induce CRC progression by activating Wnt–β-catenin signalling, and proposed this protein as a potential diagnostic and therapeutic target for this disease [38].

The objective of the present study was to analyse the expression of PRR in a series of CRC tissues and plasma from CRC patients. For this purpose, we first studied the immunohistochemical expression of PRR through the uninvolved mucosa-adenoma-CRC sequence from the same patients. In a second step, we aimed to analyse PRR expression in the centre and the infiltrating front of primary tumours but also in local lymph nodes and distant liver metastases from these primary tumours. For this reason, advanced CRCs were mainly included in the study. Finally, the soluble fraction of PRR was analysed in plasma samples from CRC patients. 

## 2. Results

### 2.1. Patients Clinical and Pathological Parameters

A set of 42 prospectively collected samples of CRC patients was employed to analyse the polyp-cancer sequence, where each case includes a tissue fraction of the three different phases of the polyp-cancer sequence: uninvolved mucosa, adenomatous polyp and cancer tissue. Males predominated in the series (34M/8F), with an average age of 68 years old. 

Additionally, we also employed a tissue collection that comprises a batch of retrospectively collected tissues from 294 advanced CRC patients with long follow-up. The collection included a tissue fraction of four different anatomic locations that represent the progression of CRC: the centre and the infiltrating front of the primary tumour, which were obtained in all the cases; tissues from local lymph node metastases, which were obtained in 232 cases; and liver metastases, obtained in 123 cases. The detailed information of the sample collection is described in Table 1.

### 2.2. PRR Protein Expression in Human Colonic Tissues

#### 2.2.1. PRR Expression According to the Gender and Age of the CRC Patients

Rho Spearman tests did not show any statistically significant correlations between gender or age and PRR protein expression levels (*p* > 0.05), which permits an unbiased study of all the cases with neither age nor gender distinction.

#### 2.2.2. PRR Expression along the Polyp-Cancer Sequence

**Figure 1:**
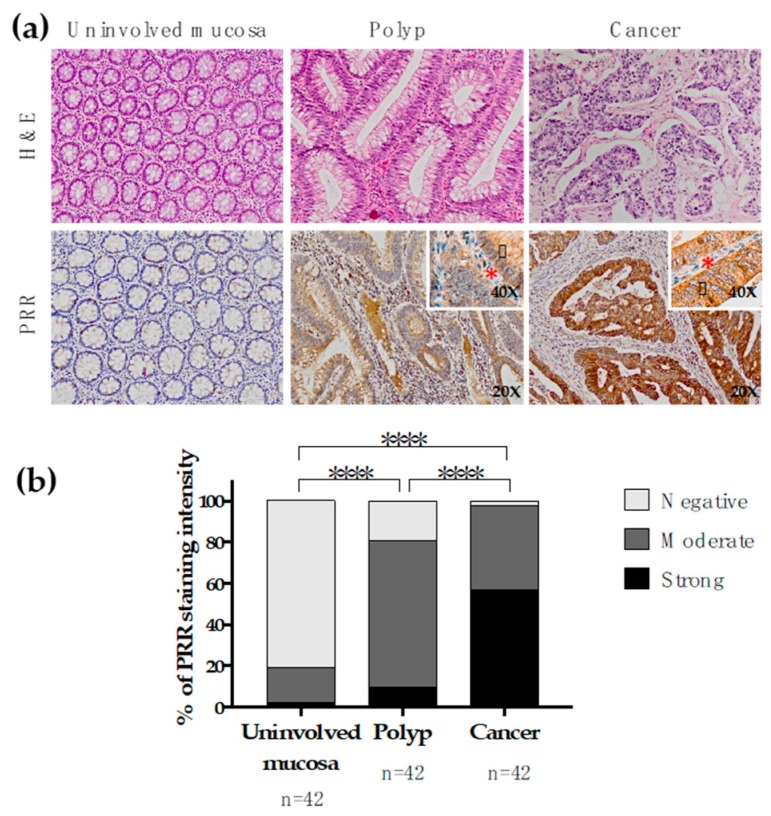
Immunohistochemical PRR staining along the adenomatous polyp-cancer sequence of CRC. (**a**) Representative fractions of uninvolved colonic mucosa, polyp and cancer tissue of 42 CRC patients were immunohistochemically stained with an antibody against PRR. Granular cytoplasmic staining was observed in neoplastic cells (black arrowhead). Stromal cellularity (lymphocytes and fibroblasts) did not express PRR (red asterisk). (**b**) PRR staining intensity was scored as negative, moderate or strong. The scores were quantified in each tissue type and statistical significance of the PRR intensity pattern among the different tissues was determined by Chi-Square test. H&E: Hematoxylin and Eosin staining. PRR: (pro)renin receptor staining.

The results disclosed that PRR is gradually augmented along the polyp-cancer sequence. In fact, PRR is absent in normal colonic tissues, while its expression rises in adenomatous polyps and strongly increases in CRC tissues (Figure 1). This gradual increase in PRR staining intensity along the polyp-cancer sequence was statistically significant when comparing the three phases among them separately (Chi square *p* < 0.00001). 

#### 2.2.3. PRR Expression According to the Histologic Subtype

We found significant PRR expression differences according to the histologic subtype. Intestinal-type adenocarcinomas (AdCs) showed significant stronger PRR expression than mucinous (MuCs) and signet ring cell carcinomas (SrcCs) (Table 2). 

To avoid histologic subtype-related bias, all the subsequent studies were conducted taking into consideration only AdCs, as they conform the great majority of all the cases.

#### 2.2.4. PRR Expression along the Conversion of the Primary Tumour into Metastasis

**Figure 2:**
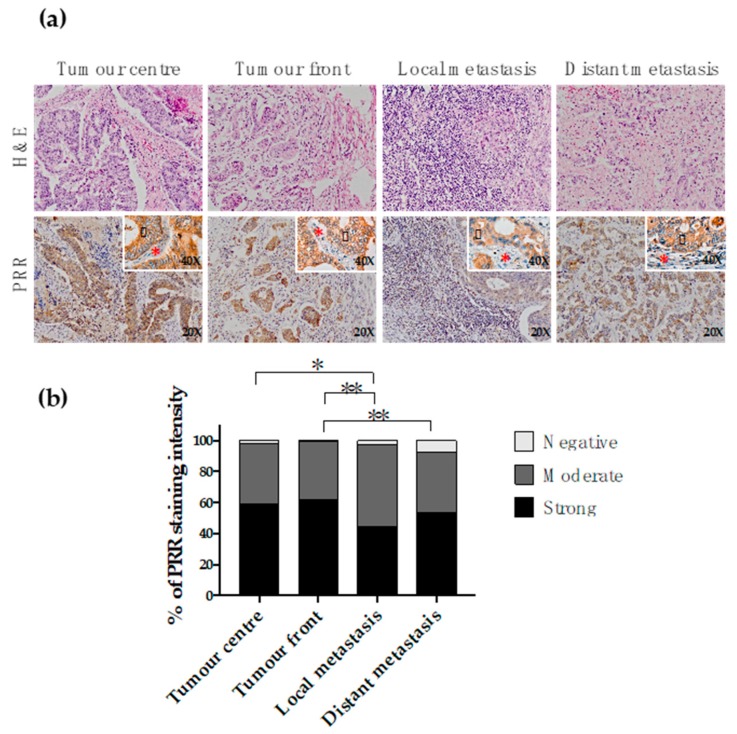
Immunohistochemical PRR staining along the conversion of the primary tumour into metastasis. (**a**) Representative fractions of the centre of the primary tumour (*n* = 228), infiltrating front of the primary tumour (*n* = 222), local lymph node metastasis (*n* = 176) and distant metastasis (*n* = 94) were immunohistochemically stained with an antibody against PRR. Granular cytoplasmic staining was observed in neoplastic cells (black arrowhead). Stromal cellularity (lymphocytes and fibroblasts) did not express PRR (red asterisk). (**b**) PRR staining intensity was scored as negative, moderate or strong. The scores were quantified in each tissue type and statistical significance of the PRR intensity pattern among the different tissues was determined by Chi-Square test. H&E: Hematoxylin and Eosin staining. PRR: (pro)renin receptor staining.

PRR staining was analysed along the progression of CRC, measuring its expression in four different locations: the centre of the primary tumour, the infiltrating front of the primary tumour, lymph node local metastasis and distant metastasis to liver (Figure 2a). The results showed that PRR is similarly expressed in both the centre and the infiltrating front of the primary tumour (Chi square *p* > 0.05), prevailing strong staining in 60% of the cases. On the other hand, both the centre and the front of the primary tumours presented stronger PRR staining than the metastatic tissues. Specifically, while PRR expression in the tumour centre was significantly stronger than the tissues obtained from local metastases (Chi square *p* = 0.021), PRR was significantly higher in the infiltrating front than in both local (Chi square *p* = 0.002) and distant metastases (Chi square *p* = 0.004) (Figure 2b).

#### 2.2.5. PRR Expression According to CRC Aggressiveness

In order to investigate whether PRR protein expression was able to predict CRC prognosis, we stratified PRR protein expression according to several clinical parameters, such as histological grade, local invasion (pT), number of affected lymph nodes (N), presence/absence of metastasis (M) and the stage (TNM system) (Table 3). 

PRR expression was stronger in tumours that presented greater invasion throughout the wall of the colon or rectum. In the tumour centre, the PRR expression of AdCs that invaded visceral peritoneum or other adjacent organs (pT4) was significantly stronger than in tumours invading muscularis propria into the pericolorectal tissues (pT3) and tumours invading submucosa and muscularis propria (pT1–pT2). Differences were also found between pT3 and pT1–pT2 tumours. In the tumour front, pT4 and pT3 tumours showed significantly stronger PRR expression than pT1–pT2.

On the other hand, PRR staining in the tumour centre was stronger in cases corresponding to N2 (metastases in four or more regional lymph nodes) compared to those belonging to N1 (metastases in 1–3 regional lymph nodes). 

In addition, PRR protein expression in primary tumours showed higher staining intensity in cases with distant metastasis at diagnosis time (M1) than those belonging to M0. It is important to note that PRR expression in lymph nodes was also stronger in the AdCs that presented distant metastases at diagnosis time (M0 vs M1, Chi-square *p* = 0.048).

Finally, PRR expression was stratified according to TNM stage. Since this series was mainly composed of advanced CRCs, stage I and II (not advanced) were grouped in a single group (stage I–II), and were compared with advanced tumours, stage III (those who had invaded lymph nodes) and IV (those who had invaded distant organs). The results showed that PRR expression in primary tumours gradually increased from low to advanced stages. Statistically significant differences were found between stage I–II and stage IV, and among stage III and IV.

No significant differences of PRR protein expression were found among samples corresponding to different histologic grades.

**Figure 3:**
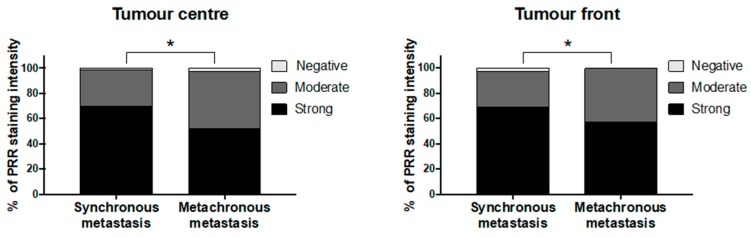
PRR protein expression according to the metastatic relapse time of CRC patients. PRR staining intensity was scored as negative, moderate or strong. The scores were quantified in each tissue group and statistical significance of the PRR intensity pattern among the different tissues was determined by Chi-Square test. (*) *p* < 0.05.

Following previously reported classifications [39], distant metastases were classified as synchronous when they were detected within the first six months from diagnosis time (*n* = 88). Those AdCs that debuted after 6 months from initial diagnosis were categorized as metachronous (*n* = 143). The results showed that PRR expression in both primary tumours’ centre and infiltrating front was significantly higher in patients with synchronous metastases compared to the ones with metachronous ones (Chi-square *p* < 0.05) (Figure 3).

#### 2.2.6. PRR Expression According to the Overall Survival of CRC Patients

**Figure 4:**
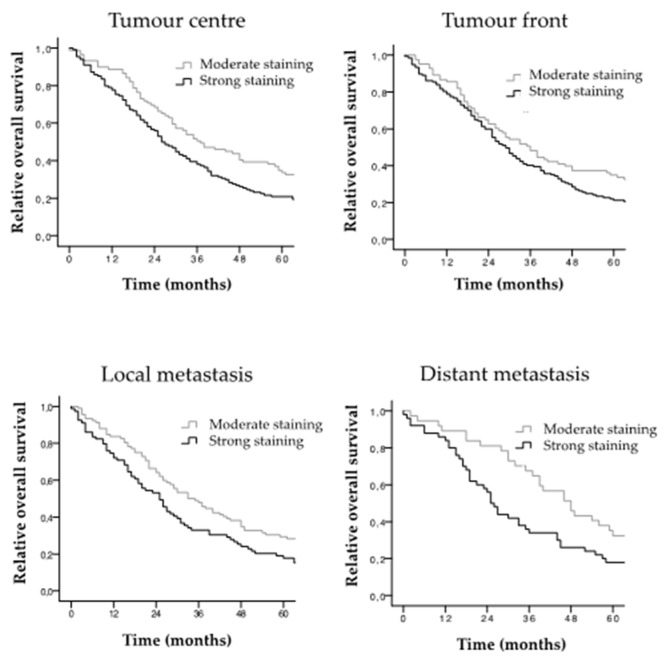
Overall survival of CRC patients according to PRR staining. Tissues scored as negative were not included in the study for conforming groups with insufficient number of cases. Survival Kaplan–Meier curves were generated along 5 years (60-months) follow-up, comparing moderate and strong PRR staining in CRC tissues belonging to the centre, infiltrating front, local metastasis and distant metastasis.

The PRR immunostaining patterns observed in the tumour centre, infiltrating front, local metastasis and distant metastasis were stratified according to patients’ 5- and 10-year overall survival (OS). The average follow-up of the AdC series (*n* = 231) was 45.01 months ranging from 0 to 184 months. Representative 60-months overall survival Kaplan–Meier curves are presented in Figure 4. In addition, 120-months overall survival curves were also performed (Supplementary Figure S1). Statistical significance at both 60- and 120-months follow-up are gathered in Table 4. 

The results showed that patients with stronger PRR protein expression in CRC tissues had worse 5- and 10-year overall survival than patients with moderate PRR expression. These results were statistically significant in the tumour centre and local and distant metastases for 5- and 10-year overall survival (Table 4).

With the aim to investigate whether PRR protein expression was an independent factor to predict CRC patients’ OS, univariate and multivariate analyses were conducted, considering several clinical and pathological variables and PRR expression. All the parameters presented statistical significance with 5-year OS (*p* < 0.05) in the univariate analysis, except for PRR protein expression in the infiltrating front of the primary tumour (*p* > 0.05) (Supplementary Table S1). All those parameters that correlated with OS in the univariate analysis were included in the Cox regression multivariate analyses (Table 5). The results indicated that PRR is not an independent prognosis factor for the prediction of CRC patients’ overall survival in none of the locations and that metastases were the main independent prognostic factor influencing 5-year survival. The same uni- and multivariate analyses were conducted at 10-year overall survival, from which similar results were obtained (Supplementary Tables S2 and S3).

We also performed a multiple logistic regression analysis to demonstrate which variable between histologic grade (G), local invasion (pT), node invasion (N) and PRR protein expression was the main factor influencing the onset of metastases. The results revealed that the most significant variable was PRR expression in the centre of the tumour (*p* < 0.01) and in the infiltrating front (*p* < 0.05). Local invasion (pT) was also included in the final step of the backward Wald method in the centre of the tumour, although it did not reach statistical significance (Table 6).

#### 2.2.7. PRR Expression According to Disease-Free Survival of CRC Patients

PRR expression was also stratified depending on the disease-free survival (DFS) of the patients. There was not any association between PRR expression in primary tumours and 5- and 10-year DFS (Supplementary Table S4).

#### 2.2.8. PRR Expression According to the Tumour Budding

With the objective to evaluate whether PRR protein expression bears any association with tumour budding, two parameters were evaluated by H&E staining: on the one hand, poorly differentiated clusters (PDCs) were evaluated. To that end, the number of the poorly differentiated clusters comprising ≥5 cancer cells were quantified (Supplementary Figure S2A). On the other hand, the desmoplastic response (DR) was assessed by classifying stroma as mature, intermediate (keloid-like collagen) or immature (myxoid stroma) (Supplementary Figure S2B). Those two parameters were then correlated with the PRR staining intensity found in the primary tumour.

The results showed no statistical significance in the correlation between CRC primary tumour PRR staining and PDC (Chi square test *p* > 0.05) and neither with DR (Chi square test *p* > 0.05) (Supplementary Table S5).

### 2.3. Soluble PRR Concentration According to CRC Aggressiveness and Patients Survival

Soluble PRR plasma concentrations were measured in the plasma of 144 CRC patients. The clinical and pathological parameters of the patients are gathered in Table 7. The plasma of 33 healthy controls were also employed, composed of 21 males and 12 females, with an average age of 57 years old (ranging from 49 to 64). Soluble PRR plasma levels follow normal distribution as proved by the Kolmogorov–Smirnov test (*p* < 0.05) and there were no sex or gender skew affecting PRR plasma levels (Rho Spearman *p* > 0.05). 

**Figure 5:**
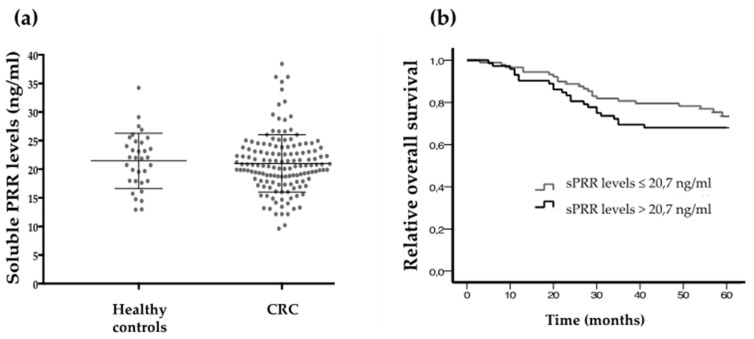
Soluble PRR concentration in plasma samples. (**a**) SPRR plasma concentrations (ng/mL) comparing healthy controls and CRC cases. (**b**) 5-year overall survival in patients expressing high and low sPRR levels (cut-off point at 20.7 ng/mL) (Log-rank test *p* = 0.271).

Statistical correlations were carried out to determine whether there is any association between soluble PRR levels and CRC aggressiveness or outcome. The results showed no association between PRR plasma concentration and histological grade, pT, N, M and stage (Table 7). Healthy controls and CRC patients also showed similar sPRR plasma levels with no statistically significant differences between them (T student test *p* > 0.05) (Figure 5a).

Patients with plasma samples presenting higher PRR levels tended to have a worse 5-year overall survival than those with lower PRR (cut-off point set at the median: 20.7 ng/mL). However, this trend did not reach statistical significance (Log-rank test *p* = 0.271) (Figure 5b)

## 3. Discussion

CRC is the consequence of several genetic and epigenetic alterations that promote gradual phenotypic changes throughout the colonic normal mucosa–adenoma–adenocarcinoma sequence [40,41,42]. Numerous proteins and signalling pathways that change along this sequence have already been identified and have been useful for the pursuit of new diagnosis/prognosis biomarkers and therapies for this disease [40,43]. Despite new molecular classifications, the treatment and prognosis of CRC patients have been unchanged during the last 20 years. However, a large number of proteomic changes still need to be elucidated for a better understanding of the behaviour of CRC and for new biomarkers and therapeutic targets identification [43].

PRR is a novel RAS receptor, the involvement of which in cancer development and progression has been under study during the last years [31,32,33,34,35,36]. Studies in different malignant tumours report the existence of PRR overexpression in cancer cells compared to non-tumoural ones. In agreement with these results, a very recent study showed a higher PRR expression in CRC cells than in normal colonic mucosa [38]. This prominent study disclosed the need for additional thorough studies that may fully elucidate the role of PRR in CRC. In this line, our study contributes to the current literature by including adenomatous precursors for a novel complete description of PRR throughout normal mucosa-adenoma-carcinoma sequence of the CRC patients. The analyses showed a gradual increase in PRR staining intensity along the sequence, which was mainly negative in the uninvolved mucosa, mostly moderate in adenomatous polyps and predominantly strong in CRC. These results reveal the idea of a potential role of this protein in the initiation and progression of CRC by the gradual acquisition of proliferative and malignant characteristics of tumour cells [38] throughout this multistep model of colorectal carcinogenesis.

This finding led us to the second objective of the study, which was to analyse PRR expression in both primary and metastatic tissues from advanced CRC patients. Thus, the second relevant result of the study was that PRR expression changed according to the histologic subtype: mucinous and signet ring cell carcinoma showed moderate PRR expression, while intestinal-type adenocarcinomas (AdC) showed predominantly stronger intensity. The first two histotypes possess a greater mucus proportion in the cell, making the dimensions of the cytoplasm smaller [44,45]. Considering that PRR staining is cytoplasmic, we hypothesize that this fact may hinder the evaluation of the staining pattern, making a hypothetical strong staining look weaker. Although this question requires further studies, we strongly suggest that these histotype-related differences in the staining pattern should be taken into consideration for the immunohistochemical evaluation of PRR in CRC tissues. 

Since TNM is the gold standard for the stratification of CRC patients into prognostic subgroups [44], we analysed whether local tumour extent (pT), invasion of regional nodes (N), metastasis occurrence (M) and TNM stage were associated with PRR expression in AdC, the most frequent CRC [44]. In primary tumours, the staining intensity of this protein gradually increased as tumour invades large intestine wall and lymph nodes. Moreover, PRR expression was significantly associated with the onset of synchronous metastases. As a consequence, stronger PRR expression was observed in cases belonging to stage IV compared to those corresponding to lower stages.

A very recent study, performed in 60 CRC primary tumours, showed similar association between PRR expression levels and tumour stage and patients’ 5-year survival [38]. Our data from a series of 231 AdC patients, that includes not only the centre and the front of primary tumours but also fractions from local and distant metastases, further support these results and reveal new information. On the one hand, strong PRR expression in primary tumours but also in metastasic tissues was associated with lower 5- and 10-year survival of CRC patients. On the other hand, multivariate analyses revealed that metastasis occurrence (M) was the most significant explanatory variable predicting survival. Besides, PRR was the most significant variable influencing metastasic onset compared to pT, pN and histologic grade. Therefore, it could be suggested that the effect of PRR expression in patients’ survival could be due to its influence on the metastasis.

Connected to this, intriguingly, we observed the loss of PRR expression in local and distant metastases compared to the primary tumours, which could suggest that although PRR seems to have an important role in the development of metastases, once it is generated, the adaptation of CRC cells in the new microenvironment might prioritise alternative signalling pathways beyond PRR-related ones (Malladi et al., 2016). Nevertheless, importantly, the expression of PRR in lymph nodes was still associated with the onset of distant metastases and a stronger expression of PRR in both nodal and hepatic metastases was correlated with lower survival of CRC patients, which suggests that the role of PRR in metastases is also related to CRC aggressiveness. The use of in vivo models in future studies and its relationship with new molecular subtypes might help clarify the specific role of PRR in metastasis occurrence, since there is no background in the literature describing it [31,32,33,34,35,36].

Recently, tumour border configuration and tumour budding have been proposed as important histomorphological variables for CRC prognosis [46,47]. The main difference between the centre and the front of the primary tumour seems to be the increased infiltrating capacity of the cells located in the tumour front [46]. Related to this, tumour budding has been defined as single cells or clusters of up to four cells with morphologic manifestations of epithelial to mesenchymal transition (EMT) at the invasive margin of CRC [47]. In this study, PRR expression was analysed in the centre and in the infiltrative front of primary tumours but we did not find any significant difference between the two locations. We neither found any correlation between PRR and the number of poorly differentiated clusters (PDC) infiltrating the tumour stroma. These results might indirectly suggest that PRR does not have a specific role in the achievement of the infiltrating capacity of CRC cells in the tumour front. However, the fact that PRR shows no heterogeneity between the centre and tumour front of the primary tumour should play in favour of a potential use of PRR as an immunohistochemical biomarker.

Thus, taken together, these results suggest a key role of PRR in CRC development and progression. The interesting study of Wang et al. demonstrated that PRR regulates proliferation and apoptosis of CRC cells in vitro and tumour growth in CRC xenografts in vivo. PRR regulated these hallmarks of cancer through Wnt–β-catenin dependent mechanisms, which is a widely recognized pathway in the initiation of CRC carcinogenesis [40].

However, although this mechanism is independent of RAS, a potential role of PRR in CRC in the context of this peptidergic system should not be ruled out. Thus, recent findings demonstrated that angiotensin-converting enzyme (ACE) and AT1R, which are the better known components and drug-targets of RAS, significantly altered their expression and activity in CRC tissues when compared to the uninvolved colorectal mucosa, and these changes were associated with CRC progression [48,49]. Our studies also demonstrated that imbalances of angiotensin receptors and angiotensin-regulating peptidases were associated with CRC development and patients’ survival [50,51,52]. These findings could explain in part the results of retrospective studies that show a protective role of RAS inhibitors in the onset of CRC [14,15,53] but also in survival rates of metastatic CRC patients [54] and have opened new perspectives in the study of colorectal carcinogenetic processes [15].

In addition to the use of PRR as a tissue biomarker for CRC detection and prognosis, we aimed to complementarily measure it in CRC patients’ plasma samples to explore its potential as a CRC biomarker in liquid biopsies. In fact, PRR is a membrane protein expressed in several tissues that, after cleavage, can be released to the extracellular space and can be detected in plasma and urine from subjects with different physiological and pathological conditions [28,30,55,56]. Shibayama et al. recently reported higher sPRR levels in patients with pancreatic ductal adenocarcinoma than in healthy controls [36]. However, our results in CRC patients’ plasma samples showed no statistically significant differences compared to healthy controls. We did not find any association between sPRR levels and aggressiveness-related parameters. 

These divergent results between solid and liquid biopsies are, however, recurrent in different studies [57,58]. For instance, in previous works, we found similar discrepancies between the expression of RAS components in tumour tissues and plasmas from CRC patients, but also detected no differences when plasma from these patients was compared to matched controls [50,51,59]. Furthermore, we conducted a similar study in samples of renal cancer patients and observed distinct results compared to CRC, concluding that changes in tumour tissue and its reflection in plasma are cancer type-dependent [60]. Therefore, further studies are necessary to understand the underlying mechanisms that might create such divergences between tissue and plasma PRR expression in CRC patients. 

Taken together, these results stress the potentiality of PRR as a CRC diagnostic/prognostic biomarker and therapeutic target based on its aggressiveness–related protein expression in human CRC tissues.

## 4. Materials and Methods 

The authors declare that all the experiments carried out in this study comply with the current Spanish and European Union legal regulations. Samples and data from patients included in this study were provided by the Basque Biobank for Research-OEHUN (www.biobancovasco.org). All patients were informed about the potential use for research of their surgically resected tissues and accepted this eventuality by signing a specific document approved by the Ethical and Scientific Committees of the Basque Country Public Health System (Osakidetza, Basque Country, Spain) (CEIC 11/51 and CEIC 18/37).

### 4.1. Immunohistochemistry

Formalin-fixed and paraffin-embedded tissues were immunostained with a rabbit polyclonal antibody specific for PRR (HPA003156; Sigma-Aldrich, St. Louis, USA) at 1/50 dilution. (https://atlasantibodies.com/products/ATP6AP2-antibody-HPA003156). The antibody’s specificity was previously tested by Western blot and immunofluorescence techniques in PRR knocked down HCT116 and SW480 cell lines (Supplementary Figure S3).

The immunostaining process was performed following routine methods in an automatic immunostainer (Dako Autostainer Plus, Dako-Agilent, Santa Barbara, USA). Briefly, antigen retrieval was carried out in a low pH buffer (K8005, Dako, Santa Barbara, USA) for 20 min at 95 °C. The samples were incubated with the primary antibody for 50 min at room temperature. Then, the primary antibody was washed, and samples were incubated for 20 min with secondary anti-rabbit antibody (K8021, Dako, Santa Barbara, USA). The EnVision-Flex detection system together with a HRP (horseradish peroxidise) enzyme-labelled polymer (SM802, Dako, Santa Barbara, USA) was employed. A positive reaction was visualized with diaminobenzydine (DAB) solution (DM827, Dako) followed by counterstaining with haematoxylin (K8008, Dako, Santa Barbara, USA).

For the staining evaluation, slides were reviewed under light microscopy. A weak granular cytoplasmic staining in the epithelial cells of renal tubules was used as external control [61]. This staining pattern was followed to score large bowel mucosa’s epithelial cell staining into negative, weak and strong.

The study of the epithelial–mesenchymal programme was made based on two histological settings. Firstly, Poorly Differentiated Clusters (PDCs) were counted, which conforms a tumour budding analogous quantification system [62,63]. For that purpose, ≥5 epithelial no gland-forming cell nests located in the tumour front were identified. After selecting the highest PDC counting area, a three-grade scoring system was followed, considering the following three groups: <5 (G1), 5–9 (G2) and ≥10 (G3) PDCs. Additionally, we analysed the stromal collagen appearance of the entire tumour, discerning three stromal desmoplastic patterns (*Desmoplastic Response* or DR): when multi-layered fine collagen fibres were noted, the stroma was considered mature; eosinophilic broad bands of collagen were described as keloid or intermediate stroma; abundant basophilic amorphous stroma was considered myxoid or immature. In all cases, the most immature grade of stroma was considered for statistical analysis [64].

The specimens were independently evaluated by two observers and discordant cases were jointly reviewed followed by a conclusive judgment.

### 4.2. ELISA Assays

In order to determine the levels of soluble PRR, a soluble (pro)renin receptor assay kit (27782; IBL) was used [55]. A volume of 100 ul of standards, reagent blank and plasma samples (1/10 dilution) were plated into a 96-well plate and incubated overnight at 4 °C. Wells were washed 4 times and 100 ul of labelled PRR antibody was added (except to the blank) and incubated for 1 h at 4 °C. Then, wells were washed 5 times and 100 uL of chromogen was added and incubated for 30 min at room temperature. A volume of 100 uL of stop solution was added to each well and the absorbance was measured at 450 nm against reagent blank. 

### 4.3. Statistical Analysis

SPSS^®^ 24.0 software was used for the statistical analysis. 

A Kolmogorov–Smirnov test was applied to data obtained from tissue and plasma samples to determine whether the numbers followed a normal distribution. Based on this information, data were analysed with parametric or non-parametric tests. We performed Pearson and Rho Spearman tests to evaluate the correlation between PRR expression and patient age and gender. T-Student and Mann–Whitney U tests (Mann-U) were used to compare tissue and plasmatic PRR levels between two groups and ANOVA and Kruskal–Wallis tests were applied to detect differences between more than two groups.

Chi-square (χ^2^) test was used to analyse the categorical PRR expression throughout the adenoma–CRC sequence, and the association with tissue PRR expression depending on pathological CRC variables. 

Finally, Kaplan–Meier curves and a log-rank test were performed to evaluate the association between the expression of tissue PRR and plasmatic PRR levels and overall survival of CRC patients. Groups were created by cut-off points based on categorical PRR expression in tissue (moderate/strong) and plasma (lower/higher than median values). Multivariate analyses were used to test the independent effects of PRR expression and clinical and pathological variables on survival (Cox regression model) and in the onset of metastasis (multiple logistic regression). 

## 5. Conclusions

The most relevant and novel findings of this study were that: (1) PRR expression gradually increases throughout the uninvolved mucosa–adenoma–CRC sequence. (2) Strong PRR expression in primary CRC tumours (AdCs) is associated with local tumour extent, nodal invasion, synchronous metastasis development, tumour stage and worse 5- and 10-year survival of CRC patients. (3) PRR expression in the centre and infiltrating front of primary tumours was similar (no intratumoural heterogeneity) and was not associated with tumour budding. (4) PRR expression in metastases was significantly lower than in primary tumours and was associated with worse survival of CRC patients. (5) Plasmatic PRR levels did not vary between CRC patients and healthy controls and were not correlated with CRC prognostic variables.

This study better supports the idea of PRR as a potential biomarker of CRC development and progression. Taking into account that this disease is a major health problem in developed countries, a better understanding of the role of PRR in colorectal carcinogenesis will be helpful for designing effective diagnostic, prognostic and therapeutic tools for CRC.

## Figures and Tables

**Table 1:** Clinical and pathological parameters of the colorectal cancer (CRC) patients’ tissues used for (pro)renin receptor (PRR) immunohistochemical analysis.

Patients’ Clinical and Pathological Data (*n* = 294)		Average (%)
Age average (range)		70 (29–93)
Follow-up months (range)		44 (0–188)
Gender	Male	203 (69%)
	Female	91 (31%)
Histologic subtype	Intestinal-type Adenocarcinoma (AdC)	231 (79%)
	Mucinous carcinoma (MuC)	50 (17%)
	Signet ring cell carcinoma (SrcC)	13 (4%)
Histologic grade (G)	G1	37 (13%)
	G2	161 (55%)
	G3	95 (32%)
Local invasion (pT)	pT1	2 (<1%)
	pT2	13 (4%)
	pT3	178 (61%)
	pT4	101 (34%)
Affected lymph nodes (N)	N0	37 (13%)
	N1	161 (55%)
	N2	95 (32%)
Distant metastases (M)	M0	182 (62%)
	M1	112 (38%)
Stage (TNM system) *	I	7 (2%)
	II	25 (9%)
	III	150 (51%)
	IV	112 (38%)

* American Joint Committee on Cancer. 7th edition.

**Table 2:** Immunohistochemical PRR staining according to CRC histologic subtypes. Representative fractions of intestinal-type, mucinous and signet ring cell carcinomas were immunohistochemically stained in the centre of the tumour, tumour front, local metastasis and distant metastasis. PRR staining intensity was scored as negative, moderate or strong. The scores were quantified in each histologic subtype and statistical significance was determined by Chi-Square test.

Heading		Negative Staining (%)	Moderate Staining (%)	Strong Staining (%)	Chi-square (*p* value)
Tumour centre	Adenocarcinoma (AdC)	2.2	39.0	58.8	9 × 10^−6^ ^a^
	Mucinous carcinoma (MuC)	4.1	75.5	20.4	
	Signet ring cell carcinoma (SrcC)	0.0	76.9	23.1	
Tumour front	Adenocarcinoma (AdC)	0.9	37.4	61.7	0.001 ^b^
	Mucinous carcinoma (MuC)	4.4	60.0	35.6	
	Signet ring cell carcinoma (SrcC)	0.0	81.8	18.2	
Local metastasis	Adenocarcinoma (AdC)	2.8	52.3	44.9	0.024 ^c^
	Mucinous carcinoma (MuC)	11.1	55.6	33.3	
	Signet ring cell carcinoma (SrcC)	0.0	84.6	15.4	
Distant metastasis	Adenocarcinoma (AdC)	7.4	39.4	53.2	0.003 ^d^
	Mucinous carcinoma (MuC)	15.8	73.7	10.5	
	Signet ring cell carcinoma (SrcC)	28.6	57.1	14.3	

^a^: Statistical significance was reached between AdCs and MuCs (*p* = 7 × 10^−6^) and AdCs and SrcCs (*p* = 0.025). ^b^: Statistical significance was reached between AdCs and MuCs (*p* = 0.002) and AdCs and SrcCs (*p* = 0.013). ^c^: Statistical significance was reached between AdCs and MuCs (*p* = 0.05). ^d^: Statistical significance was reached between AdCs and MuCs (*p* = 0.003).

**Table 3:** PRR protein expression levels according to the different pathological parameters in the centre and the infiltrating front of the analysed primary tumours.

		Tumour Centre				Tumour Front			
		Negative Staining (%)	Moderate Staining (%)	Strong Staining (%)	Chi-square (*p* value)	Negative Staining (%)	Moderate Staining (%)	Strong Staining (%)	Chi-square (*p* value)
**Grade**	**1**	2.8	41.7	55.6	0.55	0	37.5	62.5	0.270
	**2**	1.3	39.2	59.5		0.6	34.6	64.7	
	**3**	5.9	35.3	58.8		2.9	50	47.1	
**pT**	**pT1–pT2**	0	64.3	35.7	**0.001 ^a^**	0	71.4	28.6	0.051 ^b^
	**pT3**	0.7	44.7	54.7		1.4	37.2	61.4	
	**pT4**	6.3	20.3	73.4		0	30.2	69.8	
**N**	**N0**	2.7	48.6	48.6	**0.038 ^c^**	2.8	41.7	55.6	0.564
	**N1**	0	43.5	56.5		0.9	37.7	61.3	
	**N2**	4.8	28.9	66.3		0	35	65	
**M**	**M0**	3	47.8	49.3	**0.002**	0	45.4	54.6	**0.005**
	**M1**	1.1	26.6	72.3		2.2	26.1	71.7	
**Stage**	**I-II**	4.2	62.5	33.3	**0.004 ^d^**	0	52.2	47.8	**0.023 ^e^**
	**III**	2.7	44.5	52.7		0	43.9	56.1	
	**IV**	1.1	26.6	72.3		2.2	26.1	71.7	

a: Statistical significance was reached between samples belonging to pT1–T2 and pT4 (*p* = 0.004), and pT3 and pT4 (*p* = 3.8 × 10^−4^). b: Statistical significance was reached between pT1–T2 and pT3 (*p* = 0.044), and pT1–T2 and pT4 (*p* = 0.004). c: Statistical significance was reached between samples from N1 and N2 (*p* = 0.01). d: Statistical significance was reached between samples corresponding to stages I–II and IV (*p* = 0.002), and stages III and IV (*p* = 0.015). e: Statistical significance was reached between stages I–II and IV (*p* = 0.048), and stages III and IV (*p* = 0.013).

**Table 4:** Log-rank test results of the association between PRR expression in primary tumours and metastases and 5- and 10-year overall survival of CRC patients.

PRR Protein Expression	Cut-off	Follow-up Time	*n* (%)	Log-Rank (*p* value)
Tumour centre	Moderate staining/Strong staining	60 months	Alive 58 (26%) Dead 165 (74%)	**0.013**
		120 months	Alive 15 (7%) Dead 208 (93%)	**0.038**
Tumour front	Moderate staining/Strong staining	60 months	Alive 58 (26%) Dead 162 (74%)	0.051
		120 months	Alive 15 (7%) Dead 205 (93%)	0.062
Local metastasis	Moderate staining/Strong staining	60 months	Alive 41 (24%) Dead 130 (76%)	**0.029**
		120 months	Alive 14 (8%) Dead 157 (92%)	**0.038**
Distant metastasis	Moderate staining/Strong staining	60 months	Alive 21 (24%) Dead 66 (76%)	**0.019**
		120 months	Alive 3 (3%) Dead 84 (97%)	**0.037**

**Table 5:** Multivariate analysis (Cox regression model) of clinical and pathological variables and PRR expression in the centre of primary tumours and local and distant metastases for CRC patients’ overall survival prediction. Odds ratios (ORs) and inferior and superior confidence intervals (CIs) are also included. A 95% CI for OR was considered. Statistically significant values are highlighted in bold.

	Variables	Grade	pT	N	M	PRR
**Tumour Centre**	*p* value	**0.032**	**3.9 × 10^−4^**	0.777	**6.4 × 10^−5^**	0.599
	OR	1.376	1.728	1.035	1.915	1.095
	Inferior	1.028	1.277	0.818	1.392	0.780
	Superior	1.841	2.337	1.308	2.633	1.537
**Local Metastasis**	*p* value	0.087	**0.002**	0.903	**2.6 × 10^−4^**	0.089
	OR	1.334	1.689	0.980	1.939	1.358
	Inferior	0.959	1.214	0.703	1.359	0.954
	Superior	1.855	2.351	1.365	2.768	1.933
**Distant Metastasis**	*p* value	0.708	0.400	0.098	**0.021**	0.058
	OR	1.091	1.214	1.344	1.888	1.683
	Inferior	0.692	0.772	0.947	1.100	0.982
	Superior	1.720	1.909	1.909	3.242	2.882

**Table 6:** Multiple logistic regression model for the prediction of metastasis presence in CRC patients at diagnosis time. A stepwise selection procedure (backward Wald method) was used to select the final optimal model. Odds ratios (ORs) and inferior and superior confidence intervals (CIs) are also included. According to the Omnibus test, the model was statistically significant (*p* < 0.001 in the centre and *p* < 0.01 in the infiltrating front). Hosmer–Lemershow test *p* = 0.88 in the centre and *p* = 0.81 in the front). R^2^ Nagelkerke 0.1 in the centre and 0.08 in the front. A 95% CI for OR was considered. Statistically significant values are highlighted in bold.

	Multiple Logistic Regresssion					Final Step of the Wald Method	
	Variables	Grade	pT	N	PRR	PRR	pT
Tumour centre	*p* value	0.55	0.09	0.58	**0.003**	**0.002**	0.09
	B	0.24	1.36	0.25	0.87	0.92	1.35
	OR	1.28	3.9	1.28	2.39	2.5	3.85
	Inferior	0.57	0.78	0.53	1.33	1.4	0.82
	Superior	2.8	19.3	3.1	4.3	4.46	18.03
Tumour front	*p* value	0.69	0.15	0.45	**0.01**	**0.005**	-
	B	0.16	1.17	0.34	0.76	0.83	-
	OR	1.18	3.23	1.41	2.14	2.28	-
	Inferior	0.53	0.66	0.58	1.18	1.28	-
	Superior	2.61	15.8	3.43	3.89	4.08	-

**Table 7:** Clinical and pathological parameters of the CRC patients used for soluble PRR analysis and the *p* values obtained from the correlation of soluble PRR plasma levels and CRC patients’ clinical data.

Patients’ Clinical and Pathological Sata (*n* = 161)		Average (%)	sPRR Levels (ng/mL)	*p* value
Follow-up months (range)		50 (3–84)		
Age average (range)		70 (34–93)		
Gender	Male	103 (72%)		
	Female	41 (28%)		
Histologic subtype	Intestinal-type adenocarcinoma (AdC)	128 (89%)	21.12	0.331
	Mucinous carcinoma (MuC)	16 (11%)	20.22	
Histologic grade (G)	G1	6 (4%)	19.49	0.676
	G2	119 (83%)	21.16	
	G3	19 (13%)	20.60	
Local invasion (pT)	pT2	36 (25%)	20.59	0.561
	pT3	94 (65%)	20.99	
	pT4	14 (10%)	22.29	
Affected lymph nodes (N)	N0	82 (57%)	21.25	
	N1	48 (33%)	20.61	0.781
	N2	14 (10%)	21.07	
Stage (TNM system)	I	28 (19%)	20.69	
	II	53 (37%)	21.35	
	III	59 (41%)	21.18	0.296
	IV	4 (3%)	16.49	

## Supplementary Materials

The following are available online at https://www.mdpi.com/2072-6694/11/6/881/s1, Table S1: Univariate analysis (Cox regression model) of clinical and pathological variables and PRR expression for CRC patients’ 5-year overall survival prediction, Table S2: Univariate analysis (Cox regression model) of clinical and pathological variables and PRR expression for CRC patients’ 10-year overall survival prediction, Table S3: Multivariate analysis (Cox regression model) of clinical and pathological variables and PRR expression in the centre of primary tumours and local and distant metastases for CRC patients’ 10-year overall survival prediction, Table S4: Disease-free survival (DFS) of CRC patients according to PRR staining, Table S5: Association of PRR protein expression in primary tumours with tumour budding, Figure S1: 10-year overall survival of CRC patients according to PRR staining. Figure S2: Immunohistochemical H&E staining for budding evaluation in primary CRC. Figure S3: PRR knockdown in CRC cell lines for antibody’s specificity evaluation.
