# Supplementary material for: (Pro)renin Receptor Expression Increases throughout the Colorectal Adenoma—Adenocarcinoma Sequence and It Is Associated with Worse Colorectal Cancer Prognosis
Source: Cancers (Basel). 2019 Jun 24;11(6):881. doi: 10.3390/cancers11060881 (PMC6627867; doi:10.3390/cancers11060881)
Supplement: Supplementary file 1 [file cancers-11-00881-s001.zip › SUPPLEMENTARY MATERIAL/Table S3. Multivariate analysis of clinical and pathological variables and PRR expression in the centre of primary tumours and local and distant metastases for CRC patientsΓÇÖ 10-year overall survival prediction.docx]

**Table S3. Multivariate analysis (Cox regression model) of clinical and pathological variables and PRR expression in the centre of primary tumours and local and distant metastases for CRC patients’ 10-year overall survival prediction.** Odds ration (OR) and inferior and superior confidence intervals (CI) are also included. 95% CI for OR was considered. Statistically significant values are highlighted in bold.

|  | **Tumour centre** | | | | **Local metastasis** | | | | **Distant metastasis** | | | |
| --- | --- | --- | --- | --- | --- | --- | --- | --- | --- | --- | --- | --- |
| **Variables** | **p value** | **OR** | **Inferior** | **Superior** | **p value** | **OR** | **Inferior** | **Superior** | **p value** | **OR** | **Inferior** | **Superior** |
| **pT** | 0,001 | 1,580 | 1,209 | 2,067 | 0,001 | 1,672 | 1,237 | 2,258 | 0,137 | 1,320 | 0,916 | 1,904 |
| **M** | 3,3x10-4 | 1,682 | 1,267 | 2,233 | 0,001 | 1,716 | 1,239 | 2,378 | 0,051 | 1,589 | 0,998 | 2,530 |
| **PRR** | 0,654 | 1,070 | 0,796 | 1,437 | 0,079 | 1,336 | 0,967 | 1,846 | 0,116 | 1,433 | 0,915 | 2,244 |
