# Supplementary material for: (Pro)renin Receptor Expression Increases throughout the Colorectal Adenoma—Adenocarcinoma Sequence and It Is Associated with Worse Colorectal Cancer Prognosis
Source: Cancers (Basel). 2019 Jun 24;11(6):881. doi: 10.3390/cancers11060881 (PMC6627867; doi:10.3390/cancers11060881)
Supplement: Supplementary file 1 [file cancers-11-00881-s001.zip › SUPPLEMENTARY MATERIAL/Table S5. Association of PRR protein expression in primary tumours with tumour budding. .docx]

**Table S5. Association between PRR expression and tumour budding in primary tumours.** Immunohistochemically observed Poorly Differenciated Cells and Desmoplasic Response were quantified according to PRR staining intensity in the centre and front of the analysed primary tumours. Statistical significance was calculated by Chi-Square test.

|  | **Number of Poorly Differenciated Cell Clusters (PDC) in tumour front** | **Negative staining**  **(%)** | **Moderate staining**  **(%)** | **Strong staining**  **(%)** | **Chi-Square**  **(p value)** |
| --- | --- | --- | --- | --- | --- |
| PRR staining in tumour centre | 1-4 | 5,4 | 51,4 | 43,2 | 0,511 |
|  | 5-9 | 0 | 60,0 | 40,0 |  |
|  | ≥10 | 0 | 28,6 | 71,4 |  |
| PRR staining in tumour front | 1-4 | 0 | 54,3 | 45,7 | 0,140 |
|  | 5-9 | 0 | 42,9 | 57,1 |  |
|  | ≥10 | 12,5 | 37,5 | 50,0 |  |

|  | **Desmoplasic response (stroma) in tumour front** | **Negative staining**  **(%)** | **Moderate staining**  **(%)** | **Strong staining**  **(%)** | **Chi-Square**  **(p value)** |
| --- | --- | --- | --- | --- | --- |
| PRR staining in tumour centre | Mature | 0 | 53,8 | 46,2 | 0,772 |
|  | Intermediate | 6,3 | 50,0 | 43,8 |  |
|  | Myxoid | 0 | 50,0 | 50,0 |  |
| PRR staining in tumour  front | Mature | 8,3 | 50,0 | 41,7 | 0,316 |
|  | Intermediate | 0 | 53,1 | 46,9 |  |
|  | Myxoid | 0 | 38,5 | 61,5 |  |
