# Supplementary material for: (Pro)renin Receptor Expression Increases throughout the Colorectal Adenoma—Adenocarcinoma Sequence and It Is Associated with Worse Colorectal Cancer Prognosis
Source: Cancers (Basel). 2019 Jun 24;11(6):881. doi: 10.3390/cancers11060881 (PMC6627867; doi:10.3390/cancers11060881)
Supplement: Supplementary file 1 [file cancers-11-00881-s001.zip › SUPPLEMENTARY MATERIAL/Figure S1. 10-year overall survival of CRC patients according to PRR staining. .docx]

**Figure S1. 10-year overall survival of CRC patients according to PRR staining**. Survival Kaplan-Meier curves were generated along 10 years (120-months) follow-up comparing moderate and strong PRR staining in CRC tissues belonging to the centre, infiltrating front, local metastasis and distant metastasis.
