# Supplementary material for: (Pro)renin Receptor Expression Increases throughout the Colorectal Adenoma—Adenocarcinoma Sequence and It Is Associated with Worse Colorectal Cancer Prognosis
Source: Cancers (Basel). 2019 Jun 24;11(6):881. doi: 10.3390/cancers11060881 (PMC6627867; doi:10.3390/cancers11060881)
Supplement: Supplementary file 1 [file cancers-11-00881-s001.zip › SUPPLEMENTARY MATERIAL/Table S4. Disease-free survival (DFS) of CRC patients according to PRR staining.docx]

| **PRR protein expression** | **Cut-off** | **Follow-up time** | **Log-Rank**  **(p value)** |
| --- | --- | --- | --- |
| **Tumour centre** | Moderate staining / Strong staining | 5 years | 0,201 |
|  |  | 10 years | 0,332 |
| **Tumour edge** | Moderate staining / Strong staining | 5 years | 0,241 |
|  |  | 10 years | 0,217 |

**Table S4. Disease-free survival (DFS) of CRC patients according to PRR staining.** Significance was determined by Log-rank at 60 and 120 months follow-ups (Mantel-Cox).
