# Supplementary material for: (Pro)renin Receptor Expression Increases throughout the Colorectal Adenoma—Adenocarcinoma Sequence and It Is Associated with Worse Colorectal Cancer Prognosis
Source: Cancers (Basel). 2019 Jun 24;11(6):881. doi: 10.3390/cancers11060881 (PMC6627867; doi:10.3390/cancers11060881)
Supplement: Supplementary file 1 [file cancers-11-00881-s001.zip › SUPPLEMENTARY MATERIAL/Table S2. Univariate analysis of clinical and pathological variables and PRR expression for CRC patientsΓÇÖ 10-year overall survival prediction.docx]

**Table S2. Univariate analysis (Cox regression model) of clinical and pathological variables and PRR expression for CRC patients’ 10-year overall survival prediction.** Odds ratio (OR) and inferior and superior confidence intervals (CI) are also included. Statistically significant values are highlighted in bold. 95% CI for OR was considered. Statistically significant values are highlighted in bold.

| **Variables** | **p value** | **OR** | **Inferior** | **Superior** |
| --- | --- | --- | --- | --- |
| **Grade** | 0,147 | 1,204 | 0,937 | 1,547 |
| **pT** | **1,6x10^-5^** | 1,724 | 1,346 | 2,208 |
| **N** | 0,150 | 1,147 | 0,951 | 1,384 |
| **M** | **4x10^-6^** | 1,896 | 1,444 | 2,489 |
| **PRR in the centre of the primary tumour** | **0,041** | 1,338 | 1,012 | 1,769 |
| **PRR in the front of the primary tumour** | 0,066 | 1,308 | 0,983 | 1,741 |
| **PRR in the local metastasis** | **0,041** | 1,388 | 1,014 | 1,900 |
| **PRR in the distant metastasis** | **0,041** | 1,577 | 1,018 | 2,441 |
