# Supplementary material for: (Pro)renin Receptor Expression Increases throughout the Colorectal Adenoma—Adenocarcinoma Sequence and It Is Associated with Worse Colorectal Cancer Prognosis
Source: Cancers (Basel). 2019 Jun 24;11(6):881. doi: 10.3390/cancers11060881 (PMC6627867; doi:10.3390/cancers11060881)
Supplement: Supplementary file 1 [file cancers-11-00881-s001.zip › SUPPLEMENTARY MATERIAL/Table S1. Univariate analysis of clinical and pathological variables and PRR expression for CRC patientsΓÇÖ 5-year overall survival prediction.docx]

| **Variables** | **p value** | **OR** | **Inferior** | **Superior** |
| --- | --- | --- | --- | --- |
| **Grade** | **0,012** | 1,441 | 1,085 | 1,915 |
| **pT** | **10^-6^** | 1,975 | 1,498 | 2,602 |
| **N** | **0,022** | 1,284 | 1,036 | 1,592 |
| **M** | **10^-6^** | 2,161 | 1,596 | 2,927 |
| **PRR in the centre of the primary tumour** | **0,014** | 1,491 | 1,084 | 2,052 |
| **PRR in the front of the primary tumour** | 0,054 | 1,378 | 0,994 | 1,912 |
| **PRR in the local metastasis** | **0,031** | 1,459 | 1,034 | 2,060 |
| **PRR in the distant metastasis** | **0,021** | 1,801 | 1,092 | 2,972 |

**Table S1. Univariate analysis (Cox regression model) of clinical and pathological variables and PRR expression for CRC patients’ 5-year overall survival prediction.** Odds ratio (OR) and inferior and superior confidence intervals (CI) are also included. Statistically significant values are highlighted in bold. 95% CI for OR was considered. Statistically significant values are highlighted in bold.
