# Supplementary material for: (Pro)renin Receptor Expression Increases throughout the Colorectal Adenoma—Adenocarcinoma Sequence and It Is Associated with Worse Colorectal Cancer Prognosis
Source: Cancers (Basel). 2019 Jun 24;11(6):881. doi: 10.3390/cancers11060881 (PMC6627867; doi:10.3390/cancers11060881)
Supplement: Supplementary file 1 [file cancers-11-00881-s001.zip › SUPPLEMENTARY MATERIAL/Figure S2. Immunohistochemical H&E staining for budding evaluation in primary CRC.docx]

**Figure S2. Immunohistochemical H&E staining for budding evaluation in primary CRC. a:** Poorly differentiated clusters (PDC) were evaluated according to the number of cluster found in each primary tumour (red arrows). **b:** The desmoplasic response (DR) was evaluated classifying primary tumours’ stroma as mature, keloid-like or myxoid.
