# Supplementary material for: (Pro)renin Receptor Expression Increases throughout the Colorectal Adenoma—Adenocarcinoma Sequence and It Is Associated with Worse Colorectal Cancer Prognosis
Source: Cancers (Basel). 2019 Jun 24;11(6):881. doi: 10.3390/cancers11060881 (PMC6627867; doi:10.3390/cancers11060881)
Supplement: Supplementary file 1 [file cancers-11-00881-s001.zip › SUPPLEMENTARY MATERIAL/Figure S3. PRR knockdown in CRC cell lines for antibodyΓÇÖs specificity evaluation. (.docx]

**Figure S3. PRR knockdown in CRC cell lines for antibody’s specificity evaluation. (A)** HCT116 and SW480 cells were transfected with either 20 or 40nM PRR siRNA and incubated for 72h. For negative control scrambled siRNA was transfected. Western blot was used to evaluate knockdown efficiency and β-actin was used as loading control. **(B)** PRR depletion upon 40nM PRR siRNA transfection was validated by immunofluorescence. Nuclei are presented in blue (hoescht) and PRR in green. Absence of primary antibody was used as negative control.
